# Severe acute respiratory syndrome coronavirus-2 detection in domestic animals as a reservoir for the virus transmission to humans in Yogyakarta, Indonesia

**DOI:** 10.14202/vetworld.2023.341-346

**Published:** 2023-02-19

**Authors:** Yuli Purwandari Kristianingrum, Tri Untari, Asmarani Kusumawati

**Affiliations:** 1 Department of Pathology, Faculty of Veterinary Medicine, University Gadjah Mada, Yogyakarta, 55281, Indonesia; 2 Department of Microbiology, Faculty of Veterinary Medicine, University Gadjah Mada, Yogyakarta, 55281, Indonesia; 3 Department of Reproduction and Obstetrics, Faculty of Veterinary Medicine, University Gadjah Mada, Yogyakarta, 55281, Indonesia

**Keywords:** antibody, reservoir, reverse transcriptase-polymerase chain reaction, severe acute respiratory syndrome coronavirus-2

## Abstract

**Background and Aim:**

The coronavirus disease 2019 (COVID-19) is caused by severe acute respiratory syndrome coronavirus-2 (SARS-CoV-2) that attacks the respiratory and digestive tract. The SARS-CoV-2 showed systemic characteristics with various clinical symptoms from subclinical to fatal (causing death). Transmission of SARS-CoV-2 has been reported to occur from humans to pets (cats, dogs, tigers, ferrets, and poultry). Knowledge about the role of domestic animals in the transmission of SARS-CoV-2 to humans, and as reservoirs of this virus needs to be investigated further. This study aimed to detect the presence of SARS-CoV-2 in domestic animals such as dogs, cats, pigs, cows, birds, and bats that are often in contact with humans.

**Materials and Methods:**

A total of 157 samples, which included nasopharyngeal and oropharyngeal swabs, along with sera samples from domestic animals such as cats, pigs, cows, birds, and bats, were taken from Veterinary Hospitals, Veterinary Clinics, and farms around the Yogyakarta region. Detection of the virus was done using rapid detection of viral antigens, antibodies, and reverse transcriptase-polymerase chain reaction (RT-PCR) technique.

**Results:**

The results showed that 5/157 (3.1%) samples found positive against the COVID-19 virus using a rapid antibody test; however, the results were negative on the rapid antigen and RT-PCR tests. Antibody-positive samples came from animals that had a history of household COVID-19 human infection.

**Conclusion:**

Thus, findings of the present study conclude that there is a potential for transmission of the COVID-19 virus between animals and humans.

## Introduction

Coronavirus disease 2019 (COVID-19) is caused by severe acute respiratory syndrome coronavirus-2 (SARS-CoV-2). Coronavirus is a virus that tends to mutate easily and can be transmitted between different species; thus, it has the potential to be zoonotic [1]. The number of COVID-19 virus-positive cases of humans in the Yogyakarta area of Indonesia between March 2020, and September 19, 2022, were about 224,482, and the cases were continuously rising [2]. Humans are a potential source of COVID-19 transmission, not only to humans but also to animals [3]. Cases of COVID-19 virus infection in animals were drastically increased, including in cats, dogs, and horses in the USA, Europe, and Japan [4].

Transmission of SARS-CoV-2 has been reported to transmit from humans to cats [5], tigers, and lions at Bronx Zoo, New York City [6]. Cats as intermediate hosts of SARS-CoV-2 do not show obvious clinical symptoms, and their owners may not be aware that they could be a potential carrier of virus transmission to humans [7]. This incident suggests that domestic pets can be reservoirs of the virus and, have the potential to transmit the virus among themselves, between animals, and humans, even though the role of animals as a source of transmission is not yet clearly known [8].

Further, research is needed to determine the mechanism of transmission from humans to animals or vice-versa (reverse zoonosis). Transmission of virus from an infected animal to a human has been reported by a veterinarian in Thailand, was infected by COVID-19 while taking a swab sample from an infected cat [3]. Tracing, testing, and treatment (3T), method was employed to break the chain of transmission of COVID-19 in humans, but it was not routinely used for domestic animals. Thus, it is important to determine the possibility of SARS-CoV-2 transmission from domestic animals to humans. This study aimed to detect the presence of SARS-CoV-2 virus, and its characterization in domestic animals, and to determine the potential role of domestic animals as reservoirs and sources of virus transmission between species.

## Materials and Methods

### Ethical approval

All research procedures were approved by Research Ethics Commission of the Faculty of Veterinary Medicine, Universitas Gadjah Mada, Yogyakarta (No. 035/EC-FKH/Int./2022).

### Study period and location

The study was conducted from April to September 2022. The samples were collected from the animal clinics, local farms, animal markets in Yogyakarta, Indonesia. The samples were processed at the Microbiology Laboratory, Faculty of Veterinary Medicine and Center of Biotechnology at University Gadjah Mada, Yogyakarta, Indonesia.

### Animals

Total 12 dogs and 48 cats were selected for the survey that visited the animal clinics. All the selected dogs and cats had respiratory and digestive disease history. Total, 27 Pigs and 14 cows were chosen from the local farms in Yogyakarta. Further, Poultry (36) and bats (20) were taken from the animal market, and local farm in Yogyakarta. The selection of each species was based on clinical symptoms, and where animals live together with humans. The data of animals were recorded, including breed, sex, age, medical history, clinical symptoms (cough, nasal discharge, weakness, anorexia, vomiting, and diarrhea), temperature, pulse, diagnosis, and history of their owners who were positive for COVID-19 infection. The sample numbers were calculated based on clinical signs, age, sex, diagnosis, and confirmation of COVID-19 infection.

### Samples and sampling

The samples were collected between April and September 2022. Samples were taken with nasopharyngeal and oropharyngeal swabs, and blood sera. Swab samples were collected aseptically using personnel protective equipment. The specimens were collected in a vial which was supplemented with minimum essential medium added with antibiotics such as Penicillin 500 IU/mL (Meiji, Indonesia), Streptomycin 500 μg/mL (Meiji), and Nystatin 150 IU/mL (Vistapharm, Inc., Indonesia). All samples were then centrifuged at 3500× g for 15 min, and the supernatant was later stored at −70°C until further testing. Sera samples were collected in non-ethylenediaminetetraacetic acid-coated vacutainer serum tubes. The samples were properly labeled, and details of each animal, including their household COVID-19 history, were properly documented on questionnaire forms, but the samples from infected humans were not taken because of related ethical clearance. Testing of the samples was conducted at the Microbiology Laboratory, Faculty of Veterinary Medicine, Molecular Biology Laboratory, Center of Biotechnology at University of Gadjah Mada, Yogyakarta.

Early detection of virus in the animal samples (dogs, cats, cows, pigs, poultry, and bats) was carried out using rapid antigen, and antibody tests. Antigen detection method used a nasopharyngeal swab sample, and performed using a rapid antigen kit (NewLungene, Indonesia) with standard sample operational procedure. Meanwhile, antibody detection technique used a blood sample with a rapid antibody kit (Sky Test, Hangzhou Clongene Biotech Co., China). Furthermore, all samples that showed positive reactions to antigens and antibodies to COVID-19 were analyzed using reverse transcriptase-polymerase chain reaction (RT-PCR).

### RNA isolation and RT-PCR procedure

All swab samples were prepared for the detection of COVID-19, according to the protocol mentioned below. The samples in virus media were homogenized to make 200 µL for RNA extraction. The next stage is specimen preparation, RNA extraction, cDNA synthesis, and amplification using one-step reverse transcriptase PCR (Wells Bio, Inc. Republic of Korea). Each step during the PCR analysis was carried out based on the workings of the reagents. Target genes that often used are N and Ribonuclease P following those used by the Health Research Centre, Indonesia. For one RT-PCR test analysis, 5 μL of one-step RT-PCR Mix (4X) was mixed with 5 μL COVID-19 primer/probe, so total volume of the master mix was 10 μL. As much as 10 μL of master mixture was put into each quantitative PCR (qPCR) strip-tube, then added with 10 μL of RNA sample in each tube, and mixed 2–3 times (Tables-1 and 2).

**Table-1:** Concentration and purity of RNA isolation.

Sample	Purity		Concentration (ng/μL)

	A_260/230_	A_260/280_	
1	2.13	1.87	1.33
2	2.17	1.96	5.09
3	2.08	1.88	6.29
4	2.02	1.85	0.97
5	2.11	1.83	1.11

**Table-2:** Concentration and purity of cDNA.

Sample	Purity		Concentration (ng/μL)

	A_260/230_	A_260/280_	
1	1.84	2.10	801.37
2	1.85	2.15	805.28
3	1.89	2.13	840.30
4	1.82	2.22	0.97
5	1.85	2.12	806.16

Tube was inserted into the PCR machine (CFX96TM Dx System, Bio-Rad, USA). The target genes for the detection were N, and RdRP. The parameters of the PCR machine were set according to the following program: Uracil-DNA-glycosylase incubation at 25°C for 2 min; cDNA synthesis at 55°C for 10 min; pre-denaturation at 94°C for 3 min; and amplification 94°C for 15 s, and 58°C for 30 s. A total of 45 cycles were set to complete one test. Positive results were considered when the value of the signal (Ct value) 43 or less, while negative was considered when the Ct value not detected. The qPCR measures the value of amplification of cDNA quantitatively using fluorescent dyes [9].

The N (nucleocapsid) gene from all samples was detected during PCR amplification. The cDNAs were synthesized from the RNAs sample using Reverse Transcription Kit II RP1400 (Smobio, Republic of Korea). The thermal cycler conditions for cDNA synthesis were set as follows: 25°C for 10 min, 45°C for 50 min, 85°C for 5 min, and 4°C for hold during storage (Table-2). The cDNA was used as a template for the PCR. The thermal conditions for this amplification were as follows: pre-denaturation at 98°C for 45 s, denaturation at 98°C for 10 s, annealing at 56.1°C for 30 s, elongation at 72°C for 30 s, and post-elongation at 72°C for 5 min. The denaturation cycle, annealing, and elongation steps were carried out 30 times. The amplified DNA product was separated and analyzed by electrophoresis in 2% agarose gel containing ethidium bromide (0.5 µg/mL). The pair of primers used in this research was a specific primer to detect the N gene of SARS-CoV-2, which was designed using the online software *Primer3Plus* (Bioinformatics). The sequences of the primers are shown as follows: Forward primer N gene (5’ TCTAAGAAGCCTCGGCAAAA 3’) and reverse primer N gene (5’ GTGTGACTTCCATGCCAATG 3’).

### Statistical analysis

The results of nasopharyngeal and oropharyngeal swab samples using rapid antigen, antibody diagnostics, and RT-PCR technique were analyzed descriptively by looking at the results of antigen and antibody reactions and the reaction of RT-PCR values that appeared.

## Results

A total of 157 samples from dogs, cats, cows, poultry, pigs, and bats were tested for the detection of COVID-19 antibodies and antigen with a rapid detection kit. In the results, a total of five samples of cats (3) and dogs (2) were found positive for COVID-19 (Table-3). Based on the information from the animal owners showed that the animals lived with owners who were positive for COVID-19. The result of the physical examination showed that the animal had clinical symptoms of respiratory and digestive disorders (Table-4).

**Table-3:** The results of the nasal and blood swab examination.

S. No.	Type of Animal	Total	Rapid Ag	Rapid Ab	RT-PCR
1	Dog	12	0	2	0
2	Cat	48	0	3	0
3	Cow	14	0	0	0
4	Bat	20	0	0	0
5	Poultry	36	0	0	0
6	Pig	27	0	0	0
Total		157	0	5	0

**Table-4:** Clinical signs from the sampled animals.

No	Clinical sign	Total	Percentage
1	Cough	17	8.9
2	Nasal discharge	48	25
3	Lethargy	34	17.7
4	Anorexia	40	20.8
5	Diarrhea	26	13.4
6	No symptoms	27	14.2

**Figure-1:**
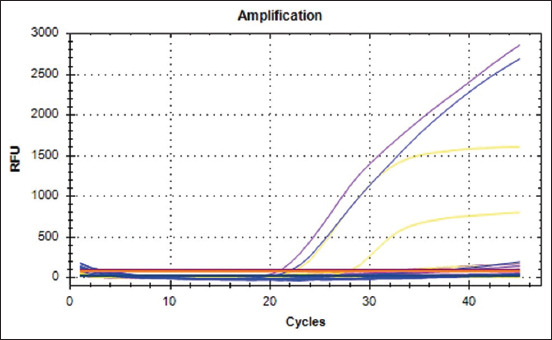
Amplification of RNA samples. the purple line shows the result of N gene, blue line shows the result of RdRP gene, and the yellow line shows the result of IC gene. All of the samples showed a negative result. The amplification result was visible only on the positive control.

**Figure-2:**
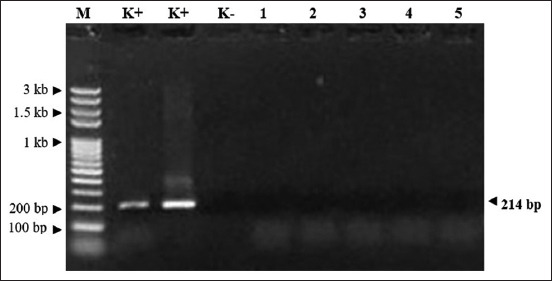
The result of electrophoresis of PCR product of N gene. M=Marker 100 bp, K+= Extracted RNA of SARS-Cov-2 from positive COVID patient as positive control (214 bp), K-=Extracted RNA of Jembrana virus as negative control, 1–5=Negative samples of swab nasal from dogs (1–2) and cats (3–5).

But the RT-PCR amplification results for COVID-19 showed negative results. Amplification curves were produced from the N gene (purple line, Ct: 19.32), and RdRP gene (blue line: Ct: 22.10) of COVID-19 positive control, the other amplification curves (yellow line) showed the result of the IC gene (Ct: 22,27). IC gene of samples showed a positive result with a Ct value of 27.85, but a negative result in the N and RdRP genes. This means that the sample of Bocil A was interpreted as a negative sample (Figure-1). The amplified RNA (214 bp) of the N gene showed a negative result (Figure-2).

## Discussion

Table-3 shows the positive COVID-19 antibody responses in dogs and cats, which represented around 3.1% of the total samples. The nasal swab sample examination with a rapid antigen test showed negative results. Based on the clinical symptoms in dogs that showed a positive reaction to the antibodies, the dogs appeared weak and anorexia, but there were no respiratory symptoms (cough and nasal discharge). The dog owners recalled that they were positive for COVID-19 with symptoms of cough, nasal discharge, and malaise. Meanwhile, antibody-positive cats showed clinical symptoms in the form of nasal discharge, lethargy, and anorexia.

Anamnesis from the cat owners showed that they were positive for COVID-19 with symptoms such as runny nose, cough, and fever. The dog and cat lived in the same house as the owners, thus created an interaction between the owners and the animals. This allowed the transmission of COVID-19 virus from humans to animals or vice-versa. Rapid transmission of SARS-CoV-2 was occurred through contact with droplets from the respiratory tract, and also through asymptomatic animals [10, 11]. The potential of the virus as a zoonotic agent, human-to-animal transmission is a very likely concern for animals and public health. Natural human-to-animal transmission of SARS-CoV-2 has been reported in COVID-19 patients in pets (dogs and cats), zoos (tigers and lions), and farms (minks) [12–14]. Furthermore, investigations of infections from SARS-CoV-2 in several animal species that are in contact with humans need to be carried out for the purposes of controlling the disease, risk factors, and determining the role of these animals in the ecology of the virus. Several researchers have shown the sensitivity of various animal species to SARS-CoV-2 through experimental studies [15, 16]. Cats, hamsters, and ferrets have a high sensitivity to SARS-CoV-2 infection and showed a variety of clinical symptoms, changes in pathology, easy transmission from natural animals, and several specific immune responses [17–19].

Based on the clinical observations, especially in the respiratory, and digestive tracts, they have shown that clinical symptoms that appear in domestic animals (dogs, cats, cows, pigs, poultry, and bats) are coughing, runny nose, weakness, loss of appetite, diarrhea, and some animals had no clinical symptoms in the respiratory, and digestive tracts. Melting from the nose (25%) and anorexia (20.8%) appeared to be the two clinical signs that affected animals the most. A positive reaction to antibodies was observed in the samples of dogs, and cats with clinical symptoms of weakness, loss of appetite, and runny nose. Many animals had symptoms of respiration disorders such as cough, discharge from the nasal, and weakness, but did not show a positive reaction to rapid antigens and antibodies. The spread of SARS-CoV-2 from human to human was the main source of transmission so the spread became more aggressive. Transmission of SARS-CoV-2 from symptomatic patients was reported to occur through droplets that came out during coughing or sneezing [20]. In addition, it has been studied that SARS-CoV-2 can be viable on aerosols (generated through a nebulizer) for at least 3 h [21].

Figure-1 depicts the amplification results of N, RdRP, and IC genes, which are represented by the respective purple, blue, and yellow lines. Figure-1 indicates that all samples showed a negative result. The amplification result was only visible in the positive control. The Ct value of the positive control was 19.38 for the N gene, 22.10 for the RdRp gene, and 22.27 for the IC gene. The Ct value of the IC gene from the sample showed a positive result with a Ct value of 27.85, but negative results in the N and RdRP genes. This indicates that the sample was interpreted as a negative sample. In addition, there were no positive results from the N gene’s RNA amplification. This suggests that animals may coexist without risk with a person who has COVID-19, even if they may have digestive or respiratory symptoms. Some clinical symptoms of diarrhea were also observed in animals which is associated with COVID-19 infection in the digestive tract. Animals infected with SARS-CoV-2 also experience infection in the digestive tract based on biopsy results on gastric epithelial cells, duodenum, and rectum. The COVID-19 virus was also detected in the feces of humans and animals.

Although the virus was not found in samples from the respiratory tract, it was found in 23% of patient feces samples. This corroborates that there is a suspicion of possible fecal-oral transmission of the COVID-19 virus [22].

Trial experiments in dogs showed a low sensitivity to SARS-CoV-2 infection, with little replication of the virus but a clear incidence of seroconversion was seen in some animals [23]. Poultry species appear to be resistant to SARS-CoV-2 infection [23, 24]. This can be seen in the results of the study that no reactions were found against antigens, and antibodies against COVID-19. Other studies have also shown the existence of several pre-clinical models for studying SARS-CoV-2. Occurrences of this virus in pigs also indicate that they are sensitive to SARS-CoV-2 infection. Pigs’ sensitivity to SARS-CoV-2 has been successfully demonstrated both experimentally and naturally [25, 26].

According to a study, total 603 dogs, and 316 cats were sampled for COVID-19 infection, and results showed that 3% of the dogs, and 6% of the cats were found COVID-19 positive with neutral antibodies. Anamnesa showed that 13% of the dogs, and 5% of the cats were from households with known COVID-19 cases. However, neither the dogs nor the cats had any respiratory complaints when the sample was taken [27]. Coronavirus disease 2019 infection in dogs and cats can occur frequently, and it is likely that infected humans transmit the virus to their pets [28]. The experimental studies showed that livestock species such as poultry, pigs, and cattle’s are not susceptible to infection [29]. Furthermore, in this study, a positive reaction of antigens and antibodies to COVID-19 was not detected in pigs and bats.

## Conclusion

The results of nasal and blood swab examinations in some domestic animals showed a positive reaction to antibodies, even though; they were negative during rapid antigens test. The present study results are supported by the case history of animal owners who were COVID-19 positive. Thus, it may be claimed that COVID-19 can spread from animals to humans.

## Authors’ Contributions

YPK and TU: Designed the study and statistical analysis. YPK, TU, and AK: Drafted and revised the manuscript. All authors have read, revised, and approved the manuscript.

## References

[1] Cutler S.J, Fooks A.R, van der Poel W.H (2010). Public health threat of new, reemerging, and neglected zoonoses in the industrialized world. Emerg. Infect. Dis.

[2] https://www.jogja.tribunnews.com/2022/09/19/update-covid-19-di-diy-19-september-2022.

[3] Sila T, Sunghan J, Laochareonsuk W.S, Kongkamol C, Ingviya T, Siripaitoon P, Kositpantawong N, Kanchanasuwan S, Hortiwakul T, Charernmak B, Nwabor O.F, Silpapojakul K, Chusri S (2022). Suspected cat-to-human transmission of SARS-CoV-2, Thailand, July-September 2021. Emerg. Infect. Dis.

[4] Haake C, Cook S, Pusterla N, Murphy B (2020). Coronavirus infections in companion animals:Virology, epidemiology, clinical and pathologic features. Viruses.

[5] Anonim^a^ (2020). Cats in NY Become the First US Pets to Test Positive for Virus AP News.

[6] Anonim^b^ (2020). United States Department of Agriculture, Animal and Plant Health Inspection Service USDA Statement on the Confirmation of COVID-19 in a tiger in New York.

[7] Halfmann P.J, Hatta M, Chiba S, Maemura T, Fan S, Takeda M, Kinoshita N, Hattori S.I, Sakai-Tagawa Y (2020). Transmission of SARS-CoV-2 in domestic cats. N. Engl. J. Med.

[8] Decaro N, Larusso A (2020). Novel human coronavirus (SARS-CoV-2):A lesson from animal coronaviruses. Vet. Microbiol.

[9] Radonic A, Thulke S, Mackay L.M, Landt O, Siegert W, Nitsche A (2004). Guideline to reference gene selection for quantitative real-time PCR. Biochem. Biophys. Res. Commun.

[10] Wu Z, McGoogan J.M (2020). Characteristics of and important lessons from the coronavirus disease (COVID-19) outbreak in China:Summary of a report of 72314 cases from the Chinese center for disease control and prevention. JAMA.

[11] ((2020)). Health Ministry of Indonesia. Information on Emerging infections of the Indonesia Health Ministry.

[12] Yoo H.S, Yoo D (2020). COVID-19 and veterinarians for one health, zoonotic-and reverse zoonotic transmissions. J. Vet. Sci.

[13] McNamara T, Richt J.A, Glickman L (2020). A critical needs assessment for research in companion animals and livestock following the pandemic of COVID-19 in humans. Vector. Borne. Zoonotic. Dis.

[14] Hernandez M, Abad D, Eiros J.M, Rodríguez-Lázaro D (2020). Are animals a Neglected transmission route of SARS-CoV-2?. Pathogens.

[15] Abdel-Moneim A.S, Abdelwhab E.M (2020). Evidence for SARS-CoV-2 infection of animal hosts. Pathogens.

[16] Sarkar J, Guha R (2020). Infectivity, virulence, pathogenicity, host-pathogen interactions of SARS and SARS-CoV-2 in experimental animals:A systematic review. Vet. Res. Commun.

[17] Halfmann P.J, Hatta M, Chiba S, Maemura T, Fan S, Takeda M, Kinoshita N, Hattori S.I, Sakai-Tagawa Y, Iwatsuki-Horimoto K, Imai M, Kawaoka Y (2020). Transmission of SARS-CoV-2 in domestic Cats. N. Engl. J. Med.

[18] Kim Y.I, Kim S.G, Kim S.M, Kim E.H, Park S.J, Yu K.M, Chang J.H, Kim E.J, Lee S, Casel M.A.B, Um J, Song M.S, Jeong H.W, Lai V.D, Kim Y, Chin S.B, Park J.S, Chung K.H, Foo S.S, Poo H, Mo I.P, Lee O.J, Webby R.J, Jung J.U, KiChoi Y (2020). Infection and Rapid transmission of SARS-CoV-2 in ferrets. Cell. Host. Microbe.

[19] Richard M, Kok A, de Meulder D, Bestebroer T.M, Lamers M.M, Okba N.M.A, van Vlissingen M.F, Rockx B, Haagmans B.L, Koopmans M.P.G, Fouchier R.A.M, Herfst S (2020). SARS-CoV-2 is transmitted via contact and via the air between ferrets. Nat. Commun.

[20] Han Y, Yang H (2020). The transmission and diagnosis of 2019 novel coronavirus infection disease (COVID-19):A Chinese perspective. J. Med. Virol.

[21] Van Doremalen N, Bushmaker T, Morris D.H, Holbrook M.G, Gamble A, Williamson BN, Tamin A, Harcourt J.L, Thornburg N.J, Gerber S.I, Lloyd-Smith J.O, de Wit E, Munster V.J (2020). Aerosol and surface stability of SARS-CoV-2 as compared with SARS-CoV-1. N. Engl. J. Med.

[22] Xiao F, Tang M, Zheng X, Liu Y, Li X, Shan H (2020). Evidence for gastrointestinal infection of SARS-CoV-2. Gastroenterology.

[23] Shi J, Wen Z, Zhong G, Yang H, Wang C, Huang B, Liu R, He X, Shuai L, Sun Z, Zhao Y, Liu P, Liang L, Cui P, Wang J, Zhang X, Guan Y, Tan W, Wu G, Chen H, Bu Z (2020). Susceptibility of ferrets, cats, dogs, and other domesticated animals to SARS-coronavirus 2. Science.

[24] Schlottau K, Rissmann M, Graaf A, Schön J, Sehl J, Wylezich C, Höper D, Mettenleiter T.C, Balkema-Buschmann A, Harder T, Grund C, Hoffmann D, Breithaupt A, Beer M ((2020)). SARS-CoV-2 in fruit bats, ferrets, pigs, and chickens:An experimental transmission study. Lancet. Microbe.

[25] Weingartl H.M, Copps J, Drebot M.A, Marszal P, Smith G, Gren J, Andonova M, Pasick J, Kitching P, Czub M (2004). Susceptibility of pigs and chickens to SARS coronavirus. Emerg. Infect. Dis.

[26] Chen W, Yan M, Yang L, Ding B, He B, Wang Y, Liu X, Liu C, Zhu H, You B, Huang S, Zhang J, Mu F, Xiang Z, Feng X, Wen J, Fang J, Yu J, Yang H, Wang J (2005). SARS-associated coronavirus transmitted from human to pig. Emerg. Infect. Dis.

[27] Patterson E.I, Elia G, Grassi A, Giordano A, Desario C, Medardo M, Smith S.L, Anderson E.R, Prince T, Patterson G.T, Lorusso E, Lucente M.S, Lanave G, Lauzi S, Bonfanti U, Stranieri A, Martella V, Basano F.S, Barrs V.R, Radford A.D, Agrimi U, Hughes G.L, Paltrinieri S, Decaro N (2020). Evidence of exposure to SARS-CoV-2 in cats and dogs from households in Italy. Nat. Commun.

[28] Leroy E.M, Ar Gouilh M, Brugere-Picoux J (2020). The risk of SARS-CoV-2 transmission to pets and other wild and domestic animals strongly mandates a one-health strategy to control the COVID-19 pandemic. One. Health.

[29] Ulrich L, Wernike K, Hofmann D, Mettenleiter T.C, Beer M (2020). Experimental infection of cattle with SARS-CoV-2. Emerg. Infect. Dis.

